# Amoxicillin Administration Regimen and Resistance Mechanisms of *Staphylococcus aureus* Established in Tissue Cage Infection Model

**DOI:** 10.3389/fmicb.2019.01638

**Published:** 2019-07-22

**Authors:** Qian Yao, Linglin Gao, Teng Xu, Yun Chen, Xin Yang, Mengmeng Han, Xiaotao He, Chengheng Li, Ruigang Zhou, Yuhui Yang

**Affiliations:** Hainan Key Laboratory of Tropical Animal Breeding and Disease Research, College of Animal Science and Technology, Hainan University, Haikou, China

**Keywords:** amoxicillin, *Staphylococcus aureus*, administration regimen, resistance mechanisms, tissue cage infection model, resistant bacteria

## Abstract

*Staphylococcus aureus* is a zoonotic pathogen that causes various life-threatening diseases. The mechanisms of action of amoxicillin against *S. aureus* are unclear. Here, we established a rabbit tissue cage infection model to evaluate the relationship between the pharmacokinetic/pharmacodynamic (PK/PD) parameters of amoxicillin and selective enrichment of resistant strains of *S. aureus* and to elucidate the evolution of its resistance to amoxicillin. *S. aureus* was injected into the tissue cages at 10^10^ colony forming units (CFU)/mL. We injected different intramuscular concentrations of amoxicillin at doses of 5, 10, 20, and 30 mg/kg body weight once a day for 5 days and 5, 10, 20, and 30 mg/kg body weight twice a day for 2.5 days. Differences in gene expression between two differentially resistant strains and a sensitive strain were evaluated using Illumina sequencing followed by COG and KEGG analysis. RT-qPCR was carried out to validate the difference in protein translation levels. Our results demonstrated that the emergence of resistant bacteria was dose dependent within a given time interval. In the same dosage group, the appearance of resistant bacteria increased with time. The resistant bacteria showed cumulative growth, and the level of resistance increased over time. The resistant bacteria were completely inhibited when the cumulative percentage of time over a 24-h period that the drug concentration exceeded the mutant prevention concentration (MPC) (%*T* > MPC) was ≥52%. We also found that *mecA* and *femX* in *S. aureus* played a leading role in the development of resistance to amoxicillin. In conclusion, it provide references for optimizing amoxicillin regimens to treat infections caused by *S. aureus*.

## Introduction

*Staphylococcus aureus* is a gram-positive bacterium that causes pneumonia, pseudomembranous colitis, pericarditis, septicemia, and other acute and chronic infections (Spaulding et al., 2012). It is found extensively in nature, and persistently colonizes around 20% of the human population. Antibiotic treatment is often ineffective because of the selective enrichment of resistant *S. aureus* strains due to the overuse and abuse of medicines (Foster et al., 2014).

As a β-lactam antibiotic, amoxicillin was introduced in human medicine in the early 1970s (Rolinson, 1974). It has also been widely used to treat infections caused by various bacteria in veterinary medicine. β-lactam antibiotic resistance exhibited by *S. aureus* is mainly due to the production of β-lactamase, which is mediated by plasmids to inhibit or hydrolyze the unique bactericidal structure of β-lactams and reduce the activity of antibacterial drugs (Keshri et al., 2018). β-lactamase confers resistance by facilitating the acquisition and stabilization of the *mecA* gene (Cohen and Sweeney, 1973; Katayama et al., 2003). In addition, the antibiotic binding sites of penicillin-binding proteins (PBPs) form a new type of binding protein, PBP 2a, which promotes the synthesis of bacterial cell walls to develop resistance (Loeffler et al., 2010). However, resistance mechanisms of *S. aureus* specifically against amoxicillin have not yet been completely elucidated.

*Staphylococcus aureus* resistance is a growing issue that poses a threat to human health caused by antimicrobial abuse (D’Costa et al., 2011). Pharmacokinetic/pharmacodynamic (PK/PD) analysis is an effective tool for evaluating optimal doses of antimicrobial agents. To prevent the emergence of resistant bacteria, many studies have evaluated the PK, PD, or PK/PD parameters of amoxicillin against *S. aureus* in order to develop a reasonable dosage regimen to slow down the rapid evolution of resistant bacteria and increase the lifespan of amoxicillin (Szultka et al., 2014; Kandeel, 2015; Velde et al., 2016). Although these studies have provided a theoretical basis, they have not explored the evolution of resistant bacteria. In this study, we exposed a standard *S. aureus* strain, ATCC6538, to different amoxicillin dosing regimens in a rabbit tissue cage infection model. We then analyzed the variability in gene expression between the resistant strains and the susceptible strains using Illumina sequencing, Clusters of orthologous groups (COG), kyoto encyclopedia of genes and genomes (KEGG), and real-time quantitative polymerase chain reaction (RT-qPCR). Our main aims were to validate the relationship between the PK/PD parameters of amoxicillin and the selective enrichment of resistant strains and to determine the genes related to resistance.

## Materials and Methods

### Animals and Ethics Statement

Forty healthy New Zealand White rabbits, including twenty females and twenty males, were selected for this experiment. The rabbits were randomly divided into nine groups of three to five rabbits each. The animals were 2–3 months old with body weights ranging from 2.05 to 3.35 kg. They received antibiotic-free fodder and water ad libitum and were housed in individual cages (the length, width, and height were 61, 43, and 50 cm, respectively). All animals were purchased from the Hainan Laboratory Animal Center, China. All animal experiments conformed to the Guide for the Care and Use of Laboratory Animals published by the United States National Institutes of Health (NIH Publication, Eighth Edition, 2011) and were approved by the Hainan University Animal Care Committee (Approval Number 2016-02; March 15, 2016).

### Bacterial Strain, Antimicrobials, and Chemicals

The *S. aureus* strain ATCC6538 was purchased from the Guangdong Huankai Microbiology Technology co., Ltd (Guangdong, China). Amoxicillin standard (98%, No. 729A022) was obtained from Beijing Solaibao Technology Co., Ltd (Beijing, China). Amoxicillin (86.2%, No. 151023) was purchased from Qilu Animal Health Products Co., Ltd (Shandong, China). Streptomycin injection (No. 20170401) was provided by North China Pharmaceutical Co., Ltd (Hebei, China). Xylazine hydrochloride injection (No. 20160801) and lidocaine injection (No. 20160501) were supplied by Shengda Animal Pharmaceutical Co., Ltd (Jilin, China).

### Measurement of MIC, MIC_99_, and MPC

The minimum inhibitory concentration (MIC) of amoxicillin against *S. aureus* was measured by the double dilution method in blank tissue cage fluid (TCF) and in Mueller-Hinton (MH) broth. A total of 11 different concentrations of the drug were prepared: 0.4 mL of liquid amoxicillin and 1.6 mL CAMHB were mixed in a test tube, and after mixing, we used the double dilution method to obtain 11 concentration gradients. Drug concentrations in the tubes 1 to 11 were 128, 64, 32, 16, 8, 4, 2, 1, 0.5, 0.25, and 0.125 μg/mL, respectively. The final volume was 1 mL in each tube. The overnight strains were adjusted approximately to 5 × 10^5^ CFU/mL and 1 mL of the inoculum was added to each tube followed by incubation at 35°C for 20 h. The minimal inhibitory concentration –that is, the lowest concentration inhibiting visible growth of the bacteria–was determined. The minimal concentration inhibiting 99% growth of the colonies after incubation at 37°C for 24 h (MIC_99_) and mutant prevention concentration (MPC) were evaluated according to the method described by Xiong et al. (2016). Stationary-phase cultures of 5 x 10^5^ CFU/mL of *S. aureus* were diluted in a concentration gradient and transferred to MH agar plates containing various drug concentrations to determine MIC_99_ values. A series of MH agar plates with the different drug concentrations were inoculated with >10^10^ colony forming units (CFU)/mL bacteria for MPC. After incubation at 37°C for 72 h without resistant strains, known as the preliminary MPC (MPCpr). For MPC, MH agar plates containing amoxicillin were produced by linearly decreasing 20% based on MPCpr. The bacteria were inoculated in the same manner. The lowest concentration that allowed no bacterial growth was the MPC. The range of MIC_99_ to MPC was the mutant selection window (MSW). Each experiment was performed in triplicate independently.

### Development of the Rabbit Tissue Cage Infection Model

Tissue cages (TC) of 43-mm diameter and 34-mL volume were autoclaved in a plastic wiffle ball. Intramuscular xylazine hydrochloride injection (0.2 mL/kg) and lidocaine injection by local infiltration anesthesia (2 mL) were used to anesthetize the rabbits. A TC was implanted subcutaneously into the back of the neck region of each animal. Following TC implantation, all rabbits received streptomycin injection (10 mg/kg) intramuscularly twice a day for 3 days. Healing incisions were applied with betadine daily for 2 weeks to avoid infection. After a month of recovery, the implanted TCs were healed into a layer of connective tissue and filled with clear TCF. Each TC absorbed about 1 mL of tissue fluid, and aseptic growth was confirmed by MH agar plate validation. Then, about 10^10^ CFU of logarithmic growth phase S. aureus suspension was concentrated in 1 mL of sterile saline solution and injected into each TC. The infection model was not established successfully until the bacterial concentration in each TC reached above 10^8^ CFU/mL after 24 h.

### Dosage Regimens and Sample Collection

Eight groups of rabbits were administered intramuscular amoxicillin at doses of 5 (*n* = 5), 10 (*n* = 5), 20 (*n* = 4), or 30 (*n* = 4) mg/kg body weight (bw) once daily for 5 days and 5 (*n* = 5), 10 (*n* = 5), 20 (*n* = 5), and 30 (*n* = 4) mg/kg bw twice daily for 2.5 days, respectively. The blank control group was treated with homogenous sterile physiological saline (*n* = 3) Xiong et al. (2016). Approximately 0.50 mL of TCF was removed from the TC at 0, 2, 4, 6, 8, 10, 12, and 24 h after each dosing for doses administered once a day and at 0, 2, 4, 6, 8, 10, and 12 h for doses administered twice a day after the first dose, the third dose, and the fifth dose. The samples were clarified by centrifugation at 3000 *g* at 4°C for 10 min and stored at −20°C prior to analysis. In addition, around 0.5 mL of sample was collected at each 12-h interval until 84 h in once daily and 168 h in twice daily. The samples were diluted serially and applied to drug-free MH agar plates or media containing 1MIC, 2MIC, 4MIC, or 8MIC of amoxicillin-containing MH agar for colony counting.

### Measurement of Amoxicillin in TCF

Amoxicillin concentration in TCF was determined by reversed-phase high-performance liquid chromatography with fluorescence detection (RP-HPLC-FLD) according to Xie et al. (2012). A C18 reverse-phase column (250 mm × 4.6 mm, internal diameter 5 μm, Thermo Fisher Scientific, United States) was used with excitation and emission wavelengths of 354 and 445 nm, respectively, at 40°C. The mobile phase consisted of 0.01 mol/L potassium dihydrogen phosphate adjusted to pH 5.5 with 0.1 mol/L potassium hydroxide (phase A) and acetonitrile (phase B) (v:v, 65:35), with a rate of 1 mL/min in the condition of invariable flow. The injection volume was 10 μL. A calibration curve was plotted from 0.05 to 4.00 μg/mL in blank TCF, with a correlation coefficient of 0.999. Recovery values ranged from 80.47 to 86.82%. The limit of determination (LOD) was 0.01 μg/mL, and the limit of quantification (LOQ) was 0.03 μg/mL. The intraday precision ranged from 7.67 to 8.61%, and the interday precision ranged from 8.82 to 10.63%. All samples were treated, including blank sample, before analysis.

To each sample (200 μL), 500 μL acetonitrile was added and vortexed for 2 min for deproteinization, and then centrifugated at 5°C, 10000 *g* for 10 min. Five-hundred microliters of the clear supernatant was mixed with 800 μL saturated dichloromethane solution and vortexed for 2 min and then centrifuged at 10000 *g* for 10 min; 100 μL of the upper water phase was transferred to 10 mL centrifuge tubes, and 200 μL of 15% trichloroacetic acid solution and 20 μL salicylaldehyde were added. The solution was vortexed for 1 min, heated in a water bath at 100°C for 45 min, and cooled to 25°C. The sample was then transferred to a 2 ml centrifuge tube. Residues were washed twice with 50% acetonitrile at a constant volume of 2 mL. After centrifugation for 15 min at 20000 *g*, the supernatant was filtered through a 0.22-μm nylon filter and then transferred to an HPLC vial. All assays were performed in triplicate.

### Analysis of Resistant Genes

To understand the regulatory mechanisms of the resistance genes, the susceptible strain, two resistant strains chosen randomly from 2MIC (low concentrations of resistant bacteria) and 8MIC (high concentrations of resistant bacteria) of amoxicillin-containing MH agar plates were cultured until the OD600 (optical density at 600 nm) reached 0.6–0.8, and then passaged five times on drug-free high salt mannitol medium agar. The sensitivity of the susceptible strain, 2MIC and 8MIC strains after passage were determined by drug-free MH agar, 1MIC, 2MIC, 4MIC, or 8MIC of amoxicillin-containing MH agar. The bacterial DNA was isolated using TAKARA kit (Beijing, China). DNA sequencing and assembly were measured by Meiyin Health Technology Co., Ltd (Beijing, China). The optimal assembly results were determined by using SOAPdenovo v2.04 splicing software. GapCloser v1.12 software was used to perform local hole filling and base correction on the assembly results. Glimmer 3.02 software was used to predict genes. Analysis of functional annotation, categorization, and protein evolution were performed by using COG database alignments. By comparing blastp with the eggNOG database, the COG annotation results corresponding to the genes could be obtained, and the proteins were functionally classified accordingly. Combined with the BLAST algorithm (blastx/blastp 2.2.28+), the obtained predicted genes were compared with the KEGG gene database (Genes), and the specific organisms involved in the corresponding genes were obtained based on the aligned KO numbers. The GO database was used to distinguish various genes according to biological function.

### Real-Time Quantitative PCR

To detect protein translation levels, total RNA was isolated by TriZOL reagent (Invitrogen) and then reverse-transcribed using the SuperScript II cDNA Synthesis Kit (TAKARA). RT-qPCR was performed by using QuantStudio TM 6 Flex SYBR^®^ Green Reagents system (Applied Biosystems, United States). Each sample was analyzed in triplicate. The internal control used for calibration of gene expression levels between samples was *GAPDH* mRNA. Analysis change between resistance strains and susceptible strain were calculated using −ΔΔCT. The primers used for PCR are shown in Table 1.

**TABLE 1:** Primers for RT-qPCR.

**Gene**	**Primer orientation**	**Nucleotide sequence**
*GAPDH*	Forward (5′-3′)	5′-TGACACTATGCAAGGTCGTTTCAC-3′
	Reverse (5′-3′)	5′-TCAGAACCGTCTAACTCTTGGTGG-3′
*mecA*	Forward (5′-3′)	5′-TACTGCTATCCACCCTCAAACA-3′
	Reverse (5′-3′)	5′-ATTTCACCTTGTCCGTAACCTG-3′
*femA*	Forward (5′-3′)	5′-AGCGTGTGTTAGTGCCTTTAGCGT-3′
	Reverse (5′-3′)	5′-CCATTGCACTGCATAACTTCCGGC-3′
*femB*	Forward (5′-3′)	5′-TTACAGAGTTAACTGTTACC-3′
	Reverse (5′-3′)	5′-ATACAAATCCAGCACGCTCT-3′
*femX*	Forward (5′-3′)	5′-TCGTGACGGTGAAGTTCAGG-3′
	Reverse (5′-3′)	5′-CACGCGTTAAGAAGCCATCG-3′

*GAPDH, glyceraldehyde-3-phosphate dehydrogenase; mecA, methicillin determinant A; femA, factor A essential for methicillin resistance; femB, factor B essential for methicillin resistance; femX, factor C essential for methicillin resistance.*

### Pharmacokinetic/Pharmacodynamic Data Analysis

Pharmacokinetic parameters including peak time (T_max_), maximum concentration (C_max_), 12 or 24 h area under the concentration–time curves (AUC_0–12h_ or AUC_0–24h_), absorption half-life (t_1/2_ka), and half-life (t_1/2_) were measured by using one compartment model of WinNonlin (version 5.2, Pharsight Corporation, Mountain View, CA, United States). Other data were calculated using Microsoft Excel.

## Results

### Results of Susceptibility Detection

The MIC and MIC_99_ values of amoxicillin against *S. aureus* ATCC6538 in the blank TCF and in MH broth were 0.25 and 0.20 μg/mL, respectively. MPCpr and MPC were 4.00 and 3.00 μg/mL, respectively. This yielded an MSW in the range of 0.20 to 3.00 μg/mL.

### Pharmacokinetic Parameters of Amoxicillin Against *S. aureus*

**FIGURE 1:**
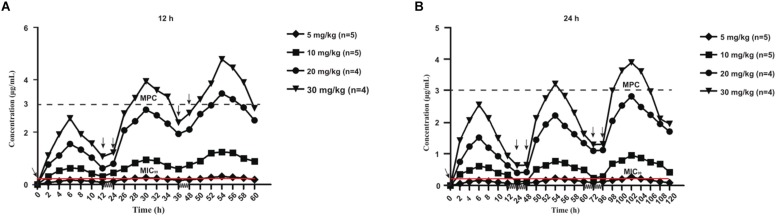
Concentration-time curves of amoxicillin against *Staphylococcus aureus* in the tissue-cage model after the first dose, the third dose, and the fifth dose. Panel **(A)** was twice daily and **(B)** was once daily dosing; n, Number of animals per group.

The mean values of amoxicillin concentration at different sampling times in each treated group were calculated and represented as eight broken lines (Figure 1). The drug concentration of 5 mg/kg bw with 12 or 24 h intervals fluctuated around the MIC_99_ value. The drug concentration of 10 mg/kg bw with 12 or 24 h intervals and 20 mg/kg bw once daily fell completely inside the MSW. The concentrations of 20 mg/kg bw twice daily and 30 mg/kg bw once daily fell around the MPC, and the 30 mg/kg bw twice daily dose was above the MPC. We also found that amoxicillin had an accumulation effect with ascending administration frequency. For example, the dose at 30 mg/kg with a 12 h interval increased the initial maximum concentration from 2.53 to 4.783 μg/mL. The pharmacokinetic parameters of amoxicillin in TF are shown in Tables 2, 3. Using the WinNonlin program, the range of AUC_0–12_ h and AUC_0–24_ h was 1.393–19.606 μg⋅h/mL and 1.916–29.421 μ⋅h/mL, respectively. The ranges of C_max_ were 0.285–4.783 μg/mL and 0.198–3.906 μg/mL, respectively. The t_1/2ka_ and t_1/2_ values were similar at 5 mg/kg and 10 mg/mg, but the parameters related to t_1/2ka_ in the 24 h group decreased significantly at 20 mg/kg and 30 mg/kg, whereas the parameters related to t_1/2_ increased significantly.

**TABLE 2:** Pharmacokinetic parameters of amoxicillin at different concentrations for twice daily administration.

**Parameters**	**5 mg/kg (n = 5)**	**10 mg/kg (n = 5)**	**20 mg/kg (n = 4)**	**30 mg/kg (n = 4)**
t_1/2ka_ (h)	3.999 ± 0.001	3.614 ± 0.028	3.342 ± 0.081	3.547 ± 0.106
t_1/2_ (h)	4.265 ± 0.012	3.964 ± 0.015	4.230 ± 0.011	3.863 ± 0.065
T_max_ (h)	5.957 ± 0.140	5.459 ± 0.132	5.412 ± 0.139	5.338 ± 0.044
C_max_ (μg/mL)	0.285 ± 0.094	1.247 ± 0.160	3.478 ± 0.028	4.783 ± 0.127
AUC_0–12__*h*_ (μg⋅h/mL)	1.393 ± 0.134	5.544 ± 0.028	12.419 ± 0.024	19.606 ± 0.075

*The t_1/2ka_, t_1/2_, T_*max*_, Cmax, and AUC_0–12__*h*_ were the mean values of multiple dose; *n*, represents the number of animals per group.*

**TABLE 3:** Pharmacokinetic parameters of amoxicillin at different concentrations for once daily administration.

**Parameters**	**5 mg/kg (n = 5)**	**10 mg/kg (n = 5)**	**20 mg/kg (n = 5)**	**30 mg/kg (*n* = 4)**
t_1/2ka_ (h)	3.859 ± 0.012	3.465 ± 0.033	2.413 ± 0.102	2.528 ± 0.013
t_1/2_ (h)	4.184 ± 0.008	4.407 ± 0.017	5.874 ± 0.020	4.871 ± 0.002
T_max_ (h)	5.796 ± 0.023	5.624 ± 0.075	5.256 ± 0.073	4.974 ± 0.521
C_max_ (μg/mL)	0.198 ± 0.016	0.960 ± 0.021	2.832 ± 0.142	3.906 ± 0.072
AUC_0–24 h_ (μg⋅h/mL)	1.916 ± 0.113	7.863 ± 0.013	18.140 ± 0.090	29.421 ± 0.570

*The t_1/2ka_, t_1/2_, T_*max*_, Cmax and AUC_0–24__*h*_ were the mean values of multiple dose; *n*, represents the number of animals per group.*

### Pharmacodynamic Parameters of Amoxicillin Against *S. aureus*

**FIGURE 2:**
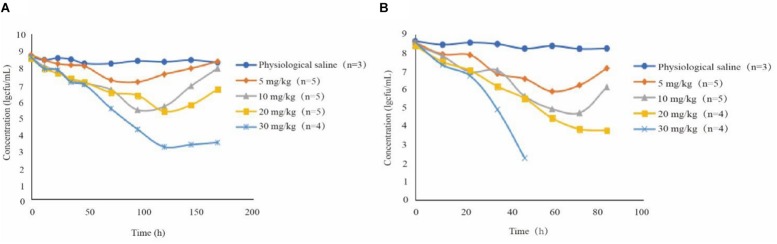
Antibacterial curve of different drug concentrations in tissue fluid for twice daily **(A)** and once daily **(B)** dosing; n, number of animals per group.

The antibacterial curve of different drug concentrations is shown in Figure 2. Bacterial population remained constant at around 10^8^ CFU/mL in the control group. In several treatment groups besides 30 mg/kg doses with 12-h intervals, the bacterial numbers non-linearly declined after 0 h. At 10 and 20 mg/kg doses with 24-h intervals and at 5 and 10 mg/kg with 12-h intervals, the bacterial numbers increased after the lowest point. In addition, the 30 mg/kg dose once daily and 20 mg/kg dose twice daily did not produce an obvious upward trend after the bacterial population dropped to 10^3^ CFU/mL. Live bacteria could not be detected even in the 30 mg/kg bw twice daily group after 60 h. The number of bacterial decreased further in groups receiving two daily doses than those receiving once daily dose under the same conditions. Amoxicillin concentration in the TC increased compared with the groups receiving two doses at the same time. For instance, in the group receiving 10 mg/kg bw with 12-h intervals, the bacterial population was around 10^7^ CFU/mL and the C_max_ of amoxicillin was approximately 1.247 μg/mL. Nevertheless, the bacterial population was around 10^6^ CFU/mL and the Cmax was nearly 0.96 μg/mL for the 10 mg/kg bw dose with 24-h intervals.

**FIGURE 3:**
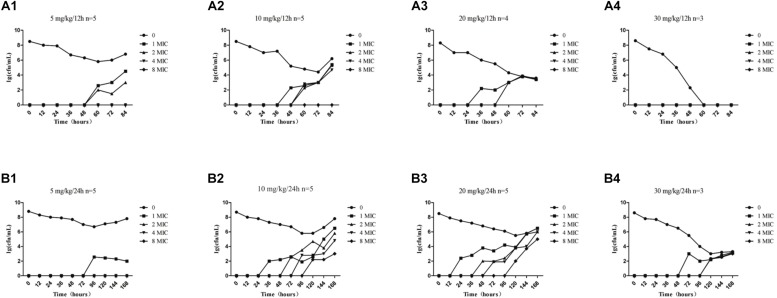
Time-killing curves of amoxicillin against *S. aureus* at different doses in the tissue-cage model. Amoxicillin at doses of 5, 10, 20, and 30 mg/kg body weight (bw) twice daily for 2.5 days **(A1–A4)** and 5, 10, 20, and 30 mg/kg bw once daily for 5 days **(B1–B4)**; n represents the number of animals per group.

The effects of administration of different drug concentrations on the composition of total bacteria and resistant bacteria in TCs are shown in Figure 3. Resistant bacteria appeared more frequently in the groups dosed at 24-h intervals than in groups dosed at 12-h intervals, except for the 5 mg/kg group. This indicates that the longer the drug concentration time falls within the MSW range, the more easily resistant bacteria can appear. Accompanied by the extension of administration time, resistant bacteria such as 1MIC, 2MIC, 4MIC, and 8MIC emerged in sequence. The numbers of resistant bacteria were equal to the total live bacteria. The low concentration resistance bacteria such as 1MIC and 2MIC appeared earlier than highly resistant bacteria such as 4MIC and 8MIC. For instance, at a dose of 10 mg/kg with 24-h intervals (Figure 3B2), 1MIC of resistant bacteria appeared at 36 h and 2MIC of resistant bacteria appeared at 72 h. Within the same drug administration interval, the longest time to emergence of resistant bacteria was in the 5 mg/kg group, and the shortest time was in the 20 mg/kg group, followed by the 10 mg/kg group. Within limits, this indicates that the higher the drug concentration, the earlier the bacterial resistance emerged. Bacterial populations decreased substantially until no bacterial re-growth at doses of 30 mg/kg with 12-h intervals (Figure 3A4).

### Relationship Between PK/PD Parameters and Resistance

**FIGURE 4:**
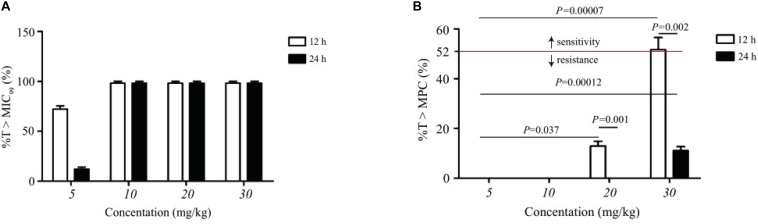
Statistic data of the relationship between resistance mutation and pharmacokinetic/pharmacodynamic parameters of amoxicillin. **(A)** Represents the effect of dose concentration on %*T* > MIC_99_ under different dosing schemes. **(B)** Represents the effect of dose concentration on %*T* > MPC under different dosing schemes. Above the dotted line indicates sensitive strain; n, number of animals per group.

The statistical values of %*T* > MIC_99_, %*T* > MPC, and resistance development of amoxicillin in TCF are presented in Figure 4. Amoxicillin is a time-dependent antimicrobial agent, and according to previous reports (Liang et al., 2011), the main concerns for the PK/PD synchronization parameters is %*T* > MPC. As shown in Figure 4, the resistant strain would not have appeared in TCs only if %*T* > MPC ≥ 52%.

### Resistant Gene Analysis

The susceptible, 2MIC and 8MIC were passaged five times and the MIC phenotype corresponded with that obtained earlier. To understand the regulatory mechanism of resistance genes, three groups of strains were cultured until the OD600 reached 0.6–0.8. By comparing blastp (BLAST 2.2.28+) with the eggNOG database, the COG annotation results corresponding to the gene were obtained. No correlation with RNA processing and repair (A), extracellular structure (W), nuclear structure (Y), cytoskeleton (Z), or other related genes were found. A gene involved in chromatin structure and kinetic function (B) was present. Unknown gene (S) and genes involved in amino acid transport and metabolism (E) were most prevalent in all three samples (Supplementary Figure S1). Samples A, B, and C genomes were BLAST-matched with the KEGG database and the regulatory network, and metabolic pathways were analyzed based on homologous localization genes. Approximately 1513 homologous genes were found in the samples. Functional gene screening using COG and other annotations related to β-lactam antibiotic resistance in the three samples. The resistance production mechanism is caused by the expression of PBP 2a protein produced by the *mecA* gene, and the fem gene regulates the synthesis of peptidoglycan in the cell wall through the functional analysis of resistance genes, which were screened based on the comparison between the resistant genes detected using the genome data of *S. aureus* and the resistant genes reported in many studies (Niemeyer et al., 1996; Sharif et al., 2009). When we compared the sequences of *mecA*, *femA*, *femB*, and *femX* genes in these three groups that were aligned using DNAman, no base substitution, misplacement, or deletion was observed, indicating that resistance did not occur at the gene level and may arise at the transcription or protein level.

### Real-Time Quantitative PCR

**FIGURE 5:**
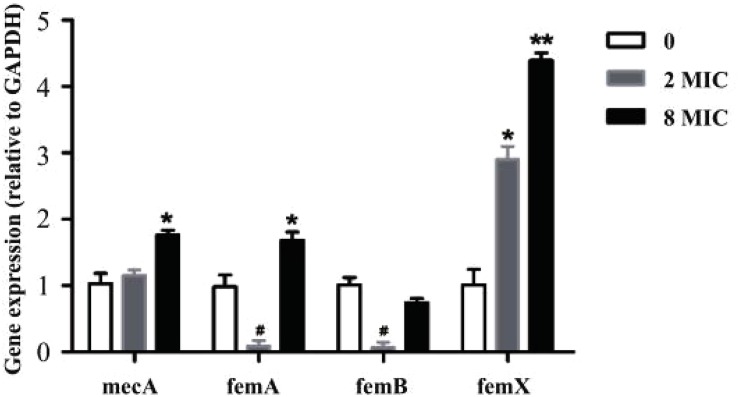
Real-time quantitative PCR results based on experiments performed on control and different resistant genes from *S. aureus*. Data are presented as fold changes between susceptible strain (white), filtrate from 2MIC (gray), and 8MIC (black) resistant strains (*n* = 3, asterisk^*^ represents upregulation, 0.01 < *p* < 0.05 and ^∗∗^*p* < 0.01, hatch mark # represents downregulation 0.01 < *p* < 0.05).

As demonstrated, amoxicillin treatment stimulated resistance development within the MSW. To verify this finding at the molecular level, the expression levels of selected genes were determined using RT-qPCR in susceptible strain, 2MIC and 8MIC strains (Figure 5). Notably, the resistance-related factors, *mecA* and *femX*, were all upregulated in 2MIC treatment and in 8MIC treatment when compared with the group with amoxicillin treatment and were dose dependent. In contrast, the expression of *femA* and *femB* was irregularly downregulated in 2MIC treatment and then upregulated in 8MIC treatment when compared with the 2MIC treatment. These results indicate that *S. aureus* likely developed resistance due to upregulated expression of the drug-resistance genes *mecA* and *femX* under treatment with amoxicillin.

## Discussion

In the present study, we found no resistant bacteria when the concentration of amoxicillin was continuously higher than the MPC with sufficient treatment time. This is a reference for clinical efficacy and evaluation of resistant bacteria production. It was reported in a study of *Escherichia coli* bacteria resistant to cefquinome in a pig tissue cage model, which showed that when %*T* > MPC was ≥50%, the occurrence of resistance could be effectively suppressed (Zhang et al., 2014). When %*T* > MPC was ≥58%, resistant bacteria could be inhibited (Xiong et al., 2016). Both amoxicillin and cefquinome are β-lactam and time-dependent drugs. In agreement with these studies, our study found that no resistant bacteria appeared when %*T* > MPC was ≥52%. In addition, our results were consistent with MSW theory, wherein resistance may occur when bacteria persisted under the drug treatment at a concentration within the MSW for a period. Combining Figure 1 with Figure 3 provides evidence that the drug concentration was proportional to the time of emergence of resistant bacteria during the same dosing interval if the drug did not completely kill the bacteria. At the same concentration, the dosing interval was positively correlated with the time of emergence of resistant bacteria.

Illumina sequencing (S1) and analysis indicated that the emergence and enrichment of resistant bacteria were related to the drug administration schedule, similar to the effects of the dosing regimen on resistant tuberculosis (Pasipanodya and Gumbo, 2011). This represents a stepwise change in the cumulative growth of resistance. Toprak et al. (2011) investigated the evolution of resistance to trimethoprim, chloramphenicol, and oxytetracycline in *E. coli*, where genome-wide sequencing of resistant *E. coli* yielded genetic variation in different resistance types. Resistance to oxytetracycline and chloramphenicol was the result of a combination of mutations in genes involved in transcription, translation, and transport. To explore the variation in the resistance mechanism of *S. aureus* under different drugs, the sensitive (S1A), 2MIC (S1B), and 8MIC strains (S1C) were selected from the plate containing antibiotics for use in whole genome sequencing and bioinformatics analyses.

In our study, *mecA*, *femA*, *femB*, and *femX* genes of resistant bacteria were screened (Figure 5). Several resistance genes were present in all three samples. The resistant gene expression levels were different between sensitive and resistant strains. These data indicate that the expression of resistance genes in the A strain may be too low. The B and C strains showed high or moderate expression levels of the resistance genes. The degree of resistance is mainly related to the expression of *mecA* and fem (Johnson et al., 1995; Menon and Nagendra, 2001; Deurenberg et al., 2010). Among these, the *mecA* gene encoding PBP 2a and the fem gene are located at different positions on the chromosome, and they play a direct or indirect role in the metabolism and structure of peptidoglycans (Niemeyer et al., 1996; Sharif et al., 2009). The results obtained from RT-qPCR (Figure 5) indicate that the expression of *mecA* was not significantly different from that in the original strain and the low-resistance strain. Nevertheless, the expression of *mecA* was significantly different between low-resistant and high-resistant bacteria (*p* > 0.05). The synthesis of normal bacterial cell walls is mainly catalyzed by normal PBP, and PBP 2a acted as a substitute for normal PBP when PBP was inhibited. Thus, upregulation of *mecA* may induce the emerge of a resistant strain.

The *S. aureus* peptidoglycan is cross-linked by a characteristic pentaglycine interpeptide bridge. Substantial genetic analyses and mutant cell wall characterization suggest that the pentaglycine interpeptide develops from the non-ribosomal peptidyl transferases sequentially (Hegde and Shrader, 2015). *FemX* employs lipid II exclusively as an acceptor for the first Gly residue (Rohrer et al., 1999; Schneider et al., 2010). *FemA* was found to catalyze the second and third glycine additions (Maidhof et al., 1991; Strandén et al., 1997). Subsequently, *femB* was presumed to add the last two glycine moieties. However, bridge formation was delayed in the *in vitro* system when all three enzymes were present (Rohrer and Berger-Bächi, 2003). The expression of *femX* was significant in the 2MIC and 8MIC resistant strains (Figure 5), which might have promoted the formation of Gly 1. Previous researches have confirmed that *femX* reacts slowly after *femA* addition, and the reaction is further impaired when *femB* is present. Conversely, lipid II conversion by *femX* may compete with non-productive binding of *femAB* (Schneider et al., 2010). This explains the much higher expression levels of *femX* than those of *femA* and *femB* in resistant strains as well as the down-regulated expression of *femA* and *femB* in 2 MIC strains and upregulation of *femX* expression. In addition, Ehlert showed that Lif and Epr can assist the insertion of serine into the interpeptide by displacing the third and fifth glycine moieties (Ehlert et al., 2000), suggesting that there may be other ways to promote the formation of pentaglycine with the downregulated expression of *femA* and *femB* in the 2MIC strain. Previous studies have shown that *S. aureus* cell wall thickening leads to increased bacterial resistance (Cui et al., 2003; Olofsson et al., 2007; Yuan et al., 2013). In summary, one of the main reasons for resistance of *S. aureus* to amoxicillin may due to the expression of *femX*, resulting in continuous repairing or abnormal thickening of cell walls, in turn promoting resistance. Other unknown genes may also help coordinate intracellular and extracellular processes to ensure that the bacteria develop resistance to antibiotics under stress.

In this study, the relationship between the PK/PD parameters of amoxicillin and the selective enrichment of resistant strains of *S. aureus* was evaluated using a rabbit TC infection model *in vivo*. Only 4 different dosage once and twice daily regimens were used to obtain the resistance prevention AUC/MIC target of %*T* > MPC ≥ 52%. It would be better if we use dose regimens that cover the entire dose-effect relationship and perform PK/PD correlation study to obtain accurate %*T* > MPC target values. Otherwise, it indicated that *mecA* and *femX* expression contribute to resistance development of *S. aureus*, and further mechanisms should be explored in future studies.

## Conclusion

The MSW of amoxicillin can affect the generation time and enrichment degree of resistant *S. aureus* strain selection. When %*T* > MPC was ≥52% of the administration interval, effective antibacterial activity was achieved, and the emergence of amoxicillin resistance was inhibited. The generation and enrichment process of resistant bacteria may be regulated by transcription and translation processes of *mecA* and *femX* genes.

## Data Availability

All data sets generated for this study are included in the manuscript and Supplemental Documentation.

## Ethics Statement

Forty healthy New Zeal and White rabbits, including twenty females and twenty males, were prepared for this experiment. All animal experiments in our study conformed to the Guide for the Care and Use of Laboratory Animals published by the United States National Institutes of Health (NIH Publication, Eighth Edition, 2011) and were approved by the Hainan University Animal Care Committee (Approval Number 2016-02; March 15, 2016).

## Author Contributions

YY and QY designed the study. LG, TX, and XY contributed to the animal management, health, and welfare analysis, and interpreted the data. YY, YC, QY, and LG drafted the work or revised it critically for the important intellectual content. QY and LG performed the experimental procedure and carried out the data validation. QY, TX, XH, MH, CL, and RZ carried out the data analysis. YY and LG wrote the manuscript. YY and YC supervised the study. YY was involved in funding acquisition.

## Conflict of Interest Statement

The authors declare that the research was conducted in the absence of any commercial or financial relationships that could be construed as a potential conflict of interest.
