# Supplementary material for: Amoxicillin Administration Regimen and Resistance Mechanisms of Staphylococcus aureus Established in Tissue Cage Infection Model
Source: Front Microbiol. 2019 Jul 22;10:1638. doi: 10.3389/fmicb.2019.01638 (PMC6662548; doi:10.3389/fmicb.2019.01638)
Supplement: Supplementary file 1 [file Data_Sheet_1.docx]

**Supplemental information**

**FIGURE S1**

COG feature annotations from sensitive strain (A) and resistance strains chosen randomly from the 2MIC (B) and 8MIC (C) of amoxicillin-containing MH agar plates.
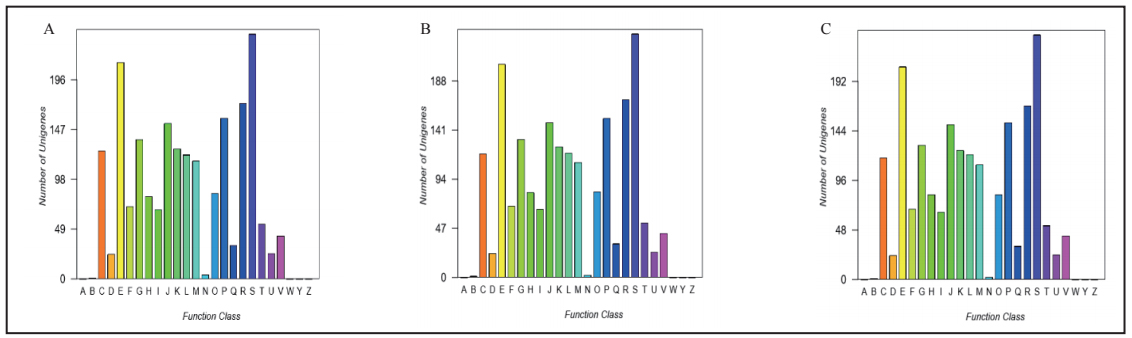
A: RNA processing and modification; B: Chromatin structure and dynamics; C: Energy production and conversion; D: Cell cycle control, cell division, and chromosome partitioning; E: Amino acid transport and metabolism; F: Nucleotide transport and metabolism; G: Carbohydrate transport and metabolism; H: Coenzyme transport and metabolism; I: Lipid transport and metabolism; J: Translation, ribosomal structure, and biogenesis; K: Transcription; L: Replication, recombination, and repair; M: Cell wall/membrane/envelope; N: Cell motility; O: Posttranslational modification, protein turnover, chaperones; P: Inorganic ion transport and metabolism; Q: Secondary metabolite biosynthesis, transport, and catabolism; R: General function prediction only; S: Function unknown; T: Signal transduction mechanisms; U: Intracellular trafficking, secretion, and vesicular transport; V: Defense mechanisms; W: Extracellular structures; Y: Nuclear structure; Z: Cytoskeleton.
